# A Survey of AI-Enabled Predictive Maintenance for Railway Infrastructure: Models, Data Sources, and Research Challenges

**DOI:** 10.3390/s26030906

**Published:** 2026-01-30

**Authors:** Francisco Javier Bris-Peñalver, Randy Verdecia-Peña, José I. Alonso

**Affiliations:** 1 Directorate of Maintenance and Conservation, Administrador de Infraestructuras Ferroviarias (ADIF), 28020 Madrid, Spain; 2 ETSI de Telecomunicación, Universidad Politécnica de Madrid, 28040 Madrid, Spain; 3 Department of R&D On-Board Communication Systems, SepsaMedha, 28320 Madrid, Spain; 4 Information Processing and Telecommunications Center, Universidad Politécnica de Madrid, 28040 Madrid, Spain

**Keywords:** artificial intelligence, railways, infrastructure, maintenance, machine learning, deep learning, digital twin, predictive maintenance, prescriptive maintenance

## Abstract

Rail transport is central to achieving sustainable and energy-efficient mobility, and its digitalization is accelerating the adoption of condition-based maintenance (CBM) strategies. However, existing maintenance practices remain largely reactive or rely on limited rule-based diagnostics, which constrain safety, interoperability, and lifecycle optimization. This survey provides a comprehensive and structured review of Artificial Intelligence techniques applied to the preventive, predictive, and prescriptive maintenance of railway infrastructure. We analyze and compare machine learning and deep learning approaches—including neural networks, support vector machines, random forests, genetic algorithms, and end-to-end deep models—applied to parameters such as track geometry, vibration-based monitoring, and imaging-based inspection. The survey highlights the dominant data sources and feature engineering techniques, evaluates the model performance across subsystems, and identifies research gaps related to data quality, cross-network generalization, model robustness, and integration with real-time asset management platforms. We further discuss emerging research directions, including Digital Twins, edge AI, and Cyber–Physical predictive systems, which position AI as an enabler of autonomous infrastructure management. This survey defines the key challenges and opportunities to guide future research and standardization in intelligent railway maintenance ecosystems.

## 1. Introduction

Rail transport has become a strategic component of global decarbonization and transport efficiency policies. The global railway market is projected to grow at a compound annual growth rate (CAGR) of 4.8% by 2030 [1]. For instance, in Asia, it is estimated that by 2035, China’s railway network will reach 200,000 km, with 90% electrification, owing to the transition toward more efficient and resilient infrastructure [2].

In the European context, the European Green Deal has acted as a catalyst for the modernization and expansion of railway infrastructure, promoting a modal shift from road transport to more sustainable modes [3]. This strategic framework has driven initiatives aligned with the development of a highly digitalized, automated, and interoperable railway system [4]. Specifically, the European Commission’s *Sustainable and Smart Mobility Strategy* insists on the need to stimulate innovation and the use of data and AI for smarter mobility, thereby reinforcing the need for these predictive strategies [5].

This momentum is reflected in the sustained growth of rail as the preferred mode of transport for freight and passengers, where rail accounts for approximately 25% of inland transport in the European Union, with a steadily increasing share [6]. This evolution reflects a structural reconfiguration of European supply chains, in which rail plays a central role in the transition toward more resilient, efficient, and low-carbon mobility, while positioning itself as a critical sector for the economy.

From both technical and operational perspectives, modern railway development is driven by two strategic imperatives: network interoperability and operational safety. Interoperability refers to the ability of multiple rail operators and infrastructure managers to run services seamlessly across different networks using harmonized procedures, unified standards, and transparent operations. In contrast, operational safety is defined as the condition in which risks arising from railway activities—whether directly associated with train movement or supporting operations—are mitigated and controlled to acceptable and regulated levels.

The achievement of these objectives is highly dependent on the effective maintenance of the various subsystems that comprise the railway infrastructure. Maintenance activities require substantial resources and coordination, and their importance has increased with the expansion of high-capacity lines, integration of digital technologies, and increasing expectations for reliability and availability of service.

In the context of increasing operational complexity and evolving performance requirements, Artificial Intelligence (AI)-enabled maintenance strategies have emerged as a pivotal approach. By using data-driven models to detect, predict, and prevent infrastructure failures, AI offers the potential to optimize resource allocation, reduce service disruptions, extend asset life cycles, and support the continuity and safety of railway operations.

### 1.1. Contributions of the Survey

This paper presents a comprehensive survey of AI techniques applied to maintenance strategies for railway infrastructure. This survey aims to encompass a broad spectrum of AI-related methodologies. The primary objective of this study is to offer an in-depth overview of AI-driven maintenance approaches, with particular emphasis on key contributions and insights that can inform and guide future research efforts.

To structure the analysis, five research questions were formulated to guide the exploration of existing studies that address critical aspects such as temporal representation, change detection, domain evolution, and their applications in railway maintenance. These questions serve as a framework for evaluating the current state-of-the-art research and identifying research gaps and opportunities.

The distinctive contribution of this study lies in its exclusive focus on fixed railway infrastructure maintenance, as opposed to broader or rolling-stock-oriented reviews. This survey synthesizes the recent literature, classifies maintenance models, and compares AI techniques in terms of their objectives, implementation strategies, and performance metrics. Furthermore, it highlights the challenges associated with data integration, model interpretability, and system scalability, while exploring innovative directions such as Digital Twins, Cyber–Physical Systems, and prescriptive analytics.

In summary, this survey makes the following contributions:

- Provides a detailed exposition of maintenance strategies, their enhancement through AI-driven algorithms, and their application to maintenance of the railway infrastructure.
- Provides a qualitative assessment of the ML and DL methodologies, serving as a practical guide for new researchers by aligning technique selection with a specific application in a railway engineering context.
- Classifies the recent literature, primarily from the past ten years, into four maintenance categories, offering a structured perspective on the state-of-the-art.
- Conducts a comparative analysis of the surveyed work, examining the AI techniques used, their objectives for improved maintenance, implementation strategies, evaluation methodologies, and associated advantages and limitations. This analysis culminates in a synthesis of key lessons learned and emerging research trends and a forward-looking discussion of future research directions.

### 1.2. Methodology

This review was conducted following the PRISMA 2020 guidelines (Preferred Reporting Items for Systematic Reviews and Meta-Analyses), which provide evidence-based recommendations to enhance the transparency and quality of systematic reviews. In Page et al. [7], detailed information and a flow diagram of the PRISMA procedure are provided.

#### 1.2.1. Objectives and Research Questions

The primary objective of this study is to analyze and synthesize scientific contributions on the application of AI in railway infrastructure maintenance procedures. This review is guided by the following research questions:

- Which is the proper and comprehensive classification of the assets (e.g., track circuit, base station of GSM-R, etc.) that comprise railway infrastructure?
- Which railway infrastructure subsystems and/or technical area components are addressed using AI-based approaches?
- Which maintenance model (e.g., preventive maintenance, predictive maintenance…) is predominant in those AI-based approaches?
- What types of data (e.g., vibrations, images, geometry) are predominantly used as input for AI-based maintenance models, and how are they acquired?
- Which AI or related techniques (e.g., neural networks, SVM, random forests, deep learning) are most frequently applied, and how do they perform comparatively across use cases?
- What are the main challenges (e.g., data quality, scalability, interpretability, integration) and emerging trends related (e.g., digital twins, federated learning) in this field?

#### 1.2.2. Search Strategy, Eligibility Criteria, and Study Selection

A comprehensive and reproducible search strategy was implemented in major scientific databases, including Scopus, Web of Science, IEEE Xplore and ScienceDirect. Manual searches of the references of the selected articles were also performed to identify additional relevant studies. The search focused on the last decade to capture the most recent advances. Thus, we included 63 studies published between 2013 and 2025 in our review. The last search was conducted on October 15, 2025. Two reviewers independently selected the titles and abstracts. Disagreements were resolved by consensus or by a third-party reviewer.

The search terms combined keywords from three conceptual axes: (i) domain (railways, railway infrastructure), (ii) task or maintenance model (preventive maintenance, predictive maintenance, prescriptive maintenance), and (iii) technology (artificial intelligence, machine learning, deep learning). The complete search strategy for the databases was as follows: *(“railway” OR “railway infrastructure”) AND (“preventive maintenance” OR “predictive maintenance” OR “prescriptive maintenance”) AND (“artificial intelligence” OR “machine learning” OR “deep learning”)*.

The inclusion criteria were (i) peer-reviewed articles (journals or conferences); (ii) studies applying AI techniques for an advanced diagnosis or prognosis of failures in fixed railway infrastructure; (iii) use of real or validated simulated data; and (iv) publications in English or Spanish.

The exclusion criteria were as follows: (i) studies focused solely on rolling stock maintenance or functional operation subsystems, without a direct infrastructure interface; (ii) literature reviews lacking novel methodology or application; (iii) opinion pieces, editorials, or non-peer-reviewed technical reports; (iv) studies using only statistical or physical methods without AI/ML components; and (v) contributions that, although technically relevant, are focused on metropolitan or light rail infrastructure.

#### 1.2.3. Data Extraction and Synthesis

A standardized template was used to extract key information from each included study: authorship, year, study objective, analyzed subsystem/component, failure type, data source/type, preprocessing techniques, AI implementation, performance metrics, main findings, and challenges.

Most of the studies reviewed employed several key metrics to evaluate the effectiveness of AI-based maintenance models. Accuracy measures the proportion of correct predictions in all classes, providing an overall indicator of the model’s reliability. In contrast, precision focuses on the correctness of positive predictions, which is particularly relevant for defect detection tasks, where false positives can lead to unnecessary interventions. The Root Mean Square Error (RMSE) quantifies the deviation between the predicted and actual values in regression-based models, which makes it essential for time-series forecasting and analysis of the degradation trend. The Area Under the Curve (AUC) is used to assess the classification performance under varying thresholds, offering a robust measure of discriminative capability in unbalanced datasets. Finally, the estimation of the Remaining Useful Life (RUL) evaluates the predictive accuracy of the models in determining the remaining operational lifespan of critical components, which is a metric of strategic importance for planning and resource optimization. Together, when available, these metrics provide a comprehensive framework for comparing AI techniques across various subsystems and maintenance objectives.

Data synthesis was conducted narratively by grouping studies by infrastructure subsystem, maintenance approach, and AI/ML technique. Summary tables were constructed to facilitate comparison and analysis, providing a structured overview of the state-of-the-art in response to the research questions.

### 1.3. Structure of the Manuscript

This manuscript is structured into seven core sections that offer a coherent and progressive exploration of the applications of AI in the maintenance of railway infrastructure. Section 2 presents a critical review of the existing literature, identifies gaps in scope and focus, and highlights the need for a comprehensive approach to the maintenance of fixed infrastructure. Section 3 introduces foundational concepts and definitions related to maintenance strategies, tracing their evolution from reactive and preventive models to predictive and prescriptive paradigms and situating AI within the broader context of Industry 5.0. Section 4 provides a theoretical overview of AI methodologies, including supervised, unsupervised, reinforcement, and hybrid learning paradigms, and clarifies their relevance to maintenance tasks. Section 5 synthesizes the practical applications of AI in key railway infrastructure subsystems (track, energy, control-command, and signaling) and classifies the reviewed studies by technique, type of data, and maintenance objective. Section 6 discusses the main challenges and strategic implications associated with the adoption of AI in railway maintenance. It highlights issues such as fragmented and low-quality data, the opacity of complex models in safety-critical contexts, and organizational barriers to system-wide integration. This section also explores the transition from isolated predictive models to holistic platforms enabled by Digital Twins and Cyber–Physical Systems, and outlines emerging trends such as prescriptive analytics, federated learning, and reinforcement learning, which are shaping the future of intelligent asset management. Section 7 concludes the manuscript by summarizing the key findings and proposing future research directions, highlighting the transformative potential of AI in enabling safer, more efficient, and resilient railway networks.

## 2. Related Work

The application of AI in the railway sector has spurred a growing body of literature reviews [8,9]. However, a detailed analysis reveals that these surveys, while valuable, exhibit significant limitations in scope and focus, leaving a significant gap in the holistic maintenance of fixed railway infrastructure [10,11].

Several reviews have focused on highly specific subdomains, offering only a partial view of the problem. For example, both Xie et al. [11] and Davari et al. [12] focus exclusively on data-driven predictive maintenance (PdM) for railway tracks. Although exhaustive within their niche, their analyses overlook other maintenance strategies (e.g., prescriptive maintenance) and omit AI applications that are not strictly based on prediction, such as expert systems or optimization algorithms. Furthermore, their scope is largely confined to track infrastructure and fails to comprehensively address energy, command-control, and signaling systems.

Other reviews have adopted an overly broad perspective, diluting the focus on infrastructure maintenance. Tang et al. [8], Besinovic et al. [9] analyzed AI across the entire train transport sector. Although providing a useful general taxonomy, their analyses encompass functional areas such as traffic management, passenger mobility, and revenue management, which inevitably reduces the depth of their treatment of infrastructure maintenance [8,9]. Similarly, ref. [10] focuses on PdM for the “railway domain,” but their final selection of articles includes a mixture of infrastructure and rolling stock components such as bogies, axles, and wheel bearings.

Some studies focus on specific enabling technologies rather than the overarching problem of infrastructure maintenance. For example, some contributions offer reviews centered on the combination of AI and Digital Twins [13,14]. Their analysis is valuable for understanding this technological synergy; however, its primary objective is not to provide an exhaustive survey of all AI applications for maintenance but rather to explore the opportunities and challenges of this specific technological pairing. Ucar et al. [15] cover a broad spectrum of AI technologies for PdM from a general industrial perspective, focusing on sectors such as automotive, energy, and manufacturing, without dedicating an exclusive and in-depth analysis to the particularities of the railway domain.

Finally, some reviews focus on infrastructure from a niche perspective. Phusakulkajorn et al. [16] concentrated on four infrastructure groups (track, catenary, civil structures, and substructure) but explicitly excluded signaling systems. Other surveys analyze AI applications through the lens of European safety and regulation, classifying works according to the structural and functional subsystems defined by EU directives. This regulatory focus, while necessary, conditions the selection and analysis, omitting applications not directly framed within that specific safety context [17].

The distinctive contribution of this study lies in its exclusive focus on fixed railway infrastructure, as opposed to broader or rolling-stock-oriented reviews. The strategic literature confirms that this focus is necessary due to the broad application scope of AI; for example, recent analyzes by UIC [18,19] identify that AI use cases are also focused on areas such as network planning & optimization, network slot allocation, traffic management, and customer care, thus reinforcing the niche focus of this survey exclusively on fixed infrastructure maintenance.

## 3. Evolution of Maintenance Models for Railway Infrastructure

According to the DIN-31051 definition, maintenance is described as a set of activities aimed at preserving and restoring the ideal condition of a system and determining and evaluating its actual condition using technical means [20].

Maintenance in railway infrastructure aims to preserve safety, availability, and life-cycle value while minimizing disruptions and costs. In practice, strategies have evolved from corrective (run-to-failure) to preventive, and more recently, to predictive and prescriptive approaches. This section defines these models and highlights their practical implications for infrastructure managers.

### 3.1. Maintenance Models

#### 3.1.1. Corrective Maintenance

Corrective maintenance, also known as run-to-failure (R2F), involves performing repairs or restoration actions only after a failure occurs [21]. Although its planning is simpler, this *reactive* strategy results in unexpected downtimes and higher breakdown costs, particularly when the asset is critical. Although this approach is straightforward, it is typically the most costly owing to unplanned downtime and potential damage [22]. Regardless of the *proactive* maintenance strategies implemented, failure is always a possibility. Therefore, it should be part of a company’s processes to establish procedures for responding to unforeseen system breakdowns or degradations. For example, in Adif, the ADIF-PE-302-001-003-SC-524 Specific Procedure regulates these operations from detection to resolution of the incident and is part of the Safety Management System (SGS).

#### 3.1.2. Preventive Maintenance

According to EN 13306 (Maintenance—Maintenance Terminology), preventive maintenance (PvM) comprises all maintenance actions performed at predetermined intervals or according to prescribed criteria, with the objective of reducing the probability of failure or degradation of asset function. EN 13306 establishes two subcategories: *predetermined maintenance* and *condition-based maintenance (CBM)*. Predetermined maintenance is executed at fixed times, mileages, or usage intervals, irrespective of the actual condition of the asset. This approach supports reliability and availability targets [23], but can also lead to unnecessary interventions or premature component replacement when degradation does not align with calendar or usage schedules [24]. In contrast, CBM schedules interventions based on the observed condition of an asset, which is obtained through inspections, measurements, or continuous monitoring. Use of indicators such as vibration, temperature, electrical parameters, or geometric deviations enables maintenance to be adapted to real asset condition, reducing over-maintenance and forming a conceptual bridge toward predictive maintenance (see Section 3.1.3). As noted in [24], CBM allows maintenance actions to be triggered when measurable deterioration occurs, improving alignment between intervention timing and actual degradation mechanisms.

**Figure 1:**
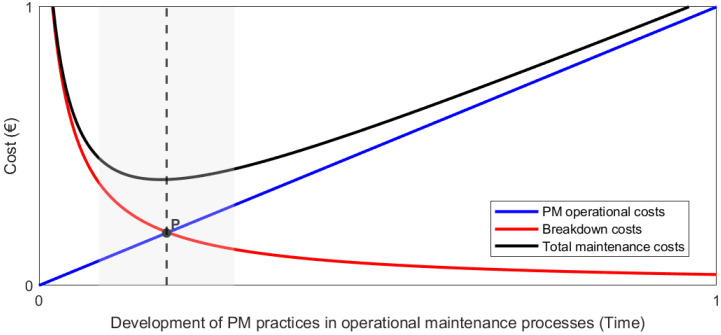
Generalization of the economical impact of PvM practices versus R2F in total maintenance costs.

As illustrated in Figure 1, selecting appropriate intervals or condition thresholds is essential to balance preventive and corrective maintenance costs.

Figure 1 shows how the development of preventive maintenance techniques leads to a reduction in breakdown or corrective maintenance costs. The challenge lies in properly adjusting the planned preventive maintenance frequencies, ideally at point ’P’ and, in any case, aiming to remain within the shaded area. In Adif’s case, these criteria are regulated by the annual approval of the General Criteria for Preventive Maintenance for each technical area. For example, in railway signaling systems, preventive maintenance can involve periodic inspection and cleaning of track circuits every six months or one year (depending on the model), regardless of their actual condition, to prevent signal failure.

#### 3.1.3. Predictive Maintenance

Predictive Maintenance (PdM) leverages real-time and historical condition data to anticipate degradation trends and determine when maintenance actions are most likely required. By complementing scheduled preventive tasks with data-driven insights, PdM supports more efficient planning, reduces unnecessary interventions, and strengthens the overall system reliability. In the railway context, PdM frameworks typically integrate sensor measurements, event logs, and diagnostic indicators into analytical models designed to detect early signs of deterioration and to forecast failure progression. Within Adif, these capabilities are being advanced through the development of *Advanced Analytics*, a strategy aimed at incorporating machine learning techniques into routine predictive workflows.

PdM is based on the principles of *condition-based maintenance* (CBM). While CBM is formally classified as preventive maintenance in EN 13306 [25], predictive maintenance expands CBM principles by incorporating prognostic models and failure forecasting capabilities, in which continuous monitoring detects deviations from expected behavior and alerts operators when the probability of deterioration increases. Unlike scheduled maintenance, CBM enables timely interventions based on the actual asset condition [26] and is widely acknowledged as a fundamental component of PdM strategies owing to its reliance on real-time diagnostic information [27]. PdM extends these capabilities by not only identifying abnormal conditions but also predicting their evolution using statistical and machine learning models.

Contemporary PdM approaches rely on advanced analytical methods that can model complex nonlinear failure mechanisms. These include convolutional neural networks (CNNs), recurrent neural networks (RNNs), autoencoders, and generative adversarial networks (GANs), which support tasks such as anomaly detection, feature extraction, and degradation forecasting. The increasing availability of high-resolution operational data, ranging from process variables and vibration signatures to event logs, has accelerated the use of data-driven frameworks for proactive maintenance decision-making [28]. Numerous studies have demonstrated that these methods can lower failure rates, reduce downtime, optimize spare parts usage, extend component life, and enhance operational safety [29,30,31].

PdM systems can incorporate a wide range of data sources and analytical paradigms, including probabilistic models, machine learning classifiers, and deep learning architectures, enabling organizations to anticipate trends and failure correlations with greater precision [32,33]. When combined with cyber–physical systems, IoT infrastructures, and cloud-based architectures, PdM solutions can operate at scale, thereby enabling real-time monitoring and collaborative decision-making. In addition, optimization models are increasingly used to balance competing objectives, such as minimizing costs, maximizing asset availability, and aligning interventions with operational constraints, thereby facilitating more intelligent maintenance planning. Overall, PdM represents a shift toward resilient, adaptive, and data-driven maintenance processes that can support the evolving demands of modern railway operations.

#### 3.1.4. Prescriptive Maintenance

**Figure 2:**
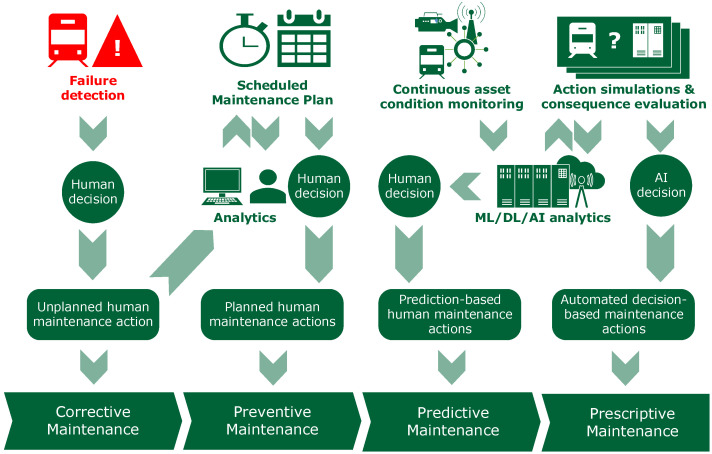
Representation of the evolution of railway infrastructure maintenance models, illustrating the shift from reactive corrective actions triggered by failure detection to scheduled preventive interventions, followed by predictive approaches based on continuous asset condition monitoring and advanced analytics, and culminating in prescriptive maintenance models that leverage simulations and consequence evaluation for automated decision-making. This progression reflects the transition from human-driven processes to intelligent, AI-based frameworks.

Prescriptive Maintenance (PsM) represents the most advanced stage in the evolution of maintenance strategies, building on the predictive capabilities of PdM to generate actionable recommendations and automated decision-making processes. The differences and relationships between PdM and PsM are illustrated in Figure 2. Unlike PdM, which forecasts when a failure might occur, PsM determines the specific actions that should be taken, when, and how to prevent failures and optimize asset performance [34].

PsM systems integrate real-time monitoring data, predictive analytics, and optimization algorithms to support intelligent maintenance planning. These systems often rely on simulation models, digital twins, and multi-objective optimization frameworks to evaluate various intervention scenarios and select the most effective strategies. In the railway domain, PsM is particularly relevant for complex infrastructure components, such as turnouts, signaling systems, and energy subsystems, where maintenance decisions must balance safety, availability, cost, and operational constraints [35].

Recent developments in AI-driven PsM include reinforcement learning, graph-based models, and context-aware human–machine interfaces. These approaches enable autonomous maintenance scheduling, resource allocation, and dynamic risk assessment. Although still emerging, PsM is expected to play a pivotal role in the transition to fully integrated and resilient railway maintenance ecosystems in the future.

### 3.2. Integration of AI Models into Maintenance Strategies in General Industry

#### 3.2.1. Machine Learning Techniques

Machine learning has emerged as a cornerstone in the development of intelligent maintenance systems that offer robust tools for pattern recognition, anomaly detection, and predictive modeling. Among the various ML algorithms applied to railway infrastructure, Random Forest [36] stands out because of its versatility and resilience in handling high-dimensional datasets, particularly when the number of features exceeds the number of observations [37]. A common challenge associated with random forests is overfitting, which is mitigated by utilizing random feature subsets to build small decision trees, resulting in a robust model suited for predictive maintenance applications [38]. Various studies have proposed its use as a prediction tool that utilizes system alarms and status information for data-driven decision-making [39]. Other studies have explored its application in the prediction of failures within naval propulsion systems [40]. The algorithm remains highly relevant in real-time applications, as demonstrated in [41], where a regression model was developed to monitor electrical faults in real-time.

One particularly notable study is that of Su [42], which introduces a failure prediction framework trained on historical data while using data sets generated by users in real-time for evaluations at query time, achieving an impressive accuracy rate of 85%.

Among other ML techniques, support vector machines (SVMs) have been extensively researched [43]. SVMs are typically used as supervised models for regression analysis and pattern recognition. For example, Praveenkumar [44] presented an SVM-based system capable of identifying faults in automobile gearboxes with remarkable accuracy exceeding 90%. Subsequent regression-based approaches have also demonstrated strong predictive performance in fault detection tasks [45]. However, one of the main challenges with SVMs is the need for optimal kernel selection, and their complex mathematical formulations often result in models that are difficult to interpret [46].

Another widely adopted algorithm in predictive maintenance is *clustering of K-means* [47,48], characterized by its unsupervised learning approach. The primary objective of K-means is to identify K distinct clusters in a data set, ensuring that closely related samples are grouped together while distant samples are assigned to separate clusters [49]. This algorithm is known for its simplicity and efficiency when working with large datasets, provided that the number of clusters remains reasonably small. Additionally, K-means supports iterative retraining, allowing cluster centers to be updated dynamically as new data samples become available.

Several studies have applied clustering techniques to define normal system behavior based on offline sensor data analysis, allowing rapid responses to anomalies [50]. More recently, Yang [51] proposed an enhanced clustering approach that improves the accuracy of anomaly detection in vehicle fault diagnostics by improving the initial cluster selection algorithm.

#### 3.2.2. Neural Networks Techniques

Artificial neural networks have shown strong performance as the foundation for PdM algorithms in many studies and datasets [31]. As a result, they have become a standard approach in industrial applications [52]. These networks are computational models inspired by the structure and function of the human brain, consisting of interconnected nodes (neurons) arranged in layers. These nodes communicate through weighted connections (synapses), which are adjusted during the training process, allowing the network to learn from the data and perform tasks such as classification, regression, and pattern recognition [53].

Among machine learning architectures frequently applied in PdM, recurrent neural networks (RNNs) and convolutional neural networks (CNNs) are particularly prominent [54]. Within the realm of RNNs, Kolokas [55] proposed an LSTM-based model to predict motor conditions by processing large-scale datasets, also comparing its performance with other machine learning algorithms. Several studies indicate that LSTM-based models demonstrate superior predictive capabilities [56].

A recent contribution by Zonta [57] highlights the ability to forecast the life expectancy of the system based on telemetry and operational data, achieving accuracy rates exceeding 92%. Other hybrid models integrate neural networks with complementary techniques, such as the study by Fahim [58], which combines non-parametric time regression (kNN) with a temporal convolutional network (TCN).

Additional approaches explore local data processing directly on embedded sensors, transmitting compact packets wirelessly to indicate the probability of system malfunction. These implementations leverage compressed recurrent neural networks [59].

In the field of CNNs for PdM, numerous contributions have emerged. Some studies focus on predicting failures in acoustic sensors. Huuhtanen and Jung [60] present a CNN-based approach for monitoring photovoltaic panel performance, predicting the daily electrical power curve as an indicator of potential malfunctions. Their numerical experiments demonstrate the ability of the model to accurately forecast the power output of operational panels. Other studies use CNNs to estimate the life expectancy of the system, such as turbofan engines, facilitating predictive and dynamic maintenance strategies for aircraft fleets while accounting for imperfect remaining useful life (RUL) forecasts [61].

A notable contribution from Samusevich, Marik, and Endel [62] describes a CNN-driven data analysis system for PdM. Once trained on telemetry datasets, the system generates precise predictions based on monitoring data. Despite the effectiveness of deep learning models, their success depends on the selection of expert data tailored to specific applications [52]. To conclude this section, we address ANN-based PsM approaches, which are based on reliable predictions to generate precise recommendations and actionable insights for maintenance activities—preventing failures before they occur [63].

Several proposals advocate novel procedural methodologies in the prescriptive maintenance planning of industrial facilities. Using simulation tools and multidimensional data analysis techniques, historical datasets—including product quality metrics, machine failure records, and production schedules—are examined. Based on identified correlations and machine input in real-time, system errors are forecast and autonomous maintenance actions are recommended without human intervention [64].

#### 3.2.3. Integration of XAI, Digital Twins, CPS, and Generative AI in Predictive Maintenance

The latest evolution of predictive maintenance strategies is increasingly shaped by the integration of Digital Twins (DTs), Cyber–Physical Systems (CPSs), Generative Artificial Intelligence (GAI), and Explainable Artificial Intelligence (XAI). This convergence enables intelligent, adaptive, and transparent maintenance frameworks that are aligned with the principles of Industry 5.0.

Digital Twins provide high-fidelity virtual representations of physical assets, allowing real-time monitoring, simulation, and lifecycle optimization. When embedded within CPS architectures, DTs facilitate continuous data exchange between the physical and digital domains, enhancing responsiveness and operational insight [13,65].

Generative AI augments the capabilities of DTs by synthesizing realistic operational data, including rare fault scenarios, thereby improving the robustness of the model and enabling proactive maintenance planning [66]. It also supports dynamic adaptation to changing system conditions and facilitates scenario-based optimizations.

Explainable AI ensures the interpretability of AI-driven decisions, which is a critical requirement in industrial contexts. By elucidating the rationale behind fault predictions and maintenance recommendations, XAI fosters trust and supports human–machine cooperation. Techniques such as LIME and SHAP have demonstrated their value in improving transparency in predictive maintenance applications [67,68,69].

Together, these technologies are expected to enable predictive maintenance systems that are more data-driven, adaptive, and interpretable; however, broader adoption requires robust validation, standardization, and governance. This integrated approach contributes to greater asset reliability, reduced operational costs, and enhanced sustainability, while also supporting spare parts optimization and human-centric decision-making [13,66,67].

## 4. Theoretical Background About AI Algorithms and Models

Machine Learning, a term originally introduced by Arthur Samuel in 1959, constitutes a subfield of Artificial Intelligence concerned with the development of algorithms that enable systems to improve their performance on a given task through experience, without the need for explicit programming [70]. ML facilitates the construction of models capable of making data-driven predictions by identifying complex patterns and structures in datasets. These models are designed to autonomously ingest new data and generate outputs, such as decisions, computations, or classifications, based on previously observed computational states.

The effectiveness of any learning paradigm relies not only on the design of the model but also on the preprocessing applied to raw data. Monitoring systems produce heterogeneous signals, such as vibration, electrical measurements, event logs, or imaging data, which require transformation before they can be used for training. Typical signal-processing operations include the Fast Fourier Transform (FFT) to extract frequency-domain features, as well as padding or windowing techniques to ensure consistent input lengths for neural architectures. For image-based inspection tasks, resizing, normalization, and related operations are essential for enforcing uniform spatial dimensions and stabilizing the training. More advanced models, such as Fully Convolutional Networks (FCNs), exploit spatial structures to enable pixel-level prediction and reduce the need for fixed-size inputs. These preprocessing steps form the foundation upon which the learning paradigms discussed in the following subsections operate.

According to [71,72,73], ML techniques have been successfully applied to a wide range of domains, including pattern and character recognition, speech recognition, computer vision, robotics, dimensionality reduction and anomaly detection, resource management and logistics, and spam detection.

The advent of artificial neural networks (ANNs), particularly deep neural networks (DNNs), commonly referred to as deep learning, marked a significant turning point in the evolution of machine learning. These architectures have achieved substantial reductions in error rates across a wide range of tasks, including image classification, speech recognition, and natural language processing. As a result, the field has increasingly adopted a dichotomous classification of methods: traditional or “classical” machine learning algorithms (e.g., decision trees, support vector machines, and logistic regression) versus neural network-based approaches, with DNNs often representing the state of the art. This shift has not only redefined performance benchmarks but has also influenced the theoretical and practical frameworks of learning paradigms in contemporary AI research.

Thus, AI-powered algorithmics encompasses a diverse spectrum of learning paradigms, each defined by the nature of the data, form of supervision, and structure of the learning task. Both Burkov [72] and Russell and Norvig [73] converge on a foundational taxonomy that includes supervised, unsupervised, semi-supervised, and reinforcement learning, while also acknowledging hybrid and symbolic paradigms, such as inductive logic programming and ensemble methods. These paradigms are distinguished by the type of feedback available to the learning agent and the assumptions made regarding the environment.

### 4.1. Supervised Learning

Supervised learning is the most studied paradigm, in which the learner is provided with a dataset of input–output pairs ${\left\{\left(x_{i},y_{i}\right)\right\}}_{i=1}^{n}$, and the goal is to learn a function f:X→Y that generalizes well to unseen data. The learning process involves minimizing a loss function L(f(x),y), such as the mean squared error for regression or cross-entropy for classification.

Formally, the empirical risk minimization (ERM) principle is often employed as follows:

(1) $$\hat{f}=\arg\min_{f\in H}\frac{1}{n}\sum_{i=1}^{n}L\left(f\left(x_{i}\right),y_{i}\right)$$

where H is the hypothesis space, emphasizing the importance of generalization bounds, such as those derived from the Probability Approximately Correct (PAC) learning framework and the bias–variance trade-off.

#### 4.1.1. Classification

In classification tasks, the output variable *y* is a categorical variable. Common algorithms include logistic regression, support vector machines (SVMs), decision trees, and neural networks. The goal was to find a decision boundary that separates the different classes. For example, in SVMs, the decision boundary is a hyperplane that maximizes the margin between the classes.

#### 4.1.2. Regression

In regression tasks, the output variable *y* is continuous. Algorithms such as linear regression, ridge regression, and neural networks are used to model the relationship between the input features and continuous output. The objective was to minimize the mean squared error between the predicted and actual values.

### 4.2. Unsupervised Learning

In unsupervised learning, the learner is given a dataset ${\left\{x_{i}\right\}}_{i=1}^{n}$ without the corresponding labels. The objective is to uncover hidden structures in the data, such as clusters, manifolds, or latent variables. Common tasks include:

#### 4.2.1. Clustering

Clustering involves partitioning data into groups $C_{1},\ldots ,C_{k}$ such that the intra-cluster similarity is maximized. Algorithms such as k-means, hierarchical clustering, and Density-Based Spatial Clustering of Applications with Noise (DBSCAN) are commonly used. For instance, this last is a popular unsupervised machine learning method that groups together points that are closely packed (i.e., have many nearby neighbors), while marking points that lie alone in low-density regions as outliers or noise.

#### 4.2.2. Dimensionality Reduction

The reduction in dimensionality aims to find a mapping x↦z where $z\in R^{d}$, d≪dim(x), preserves the essential structure. Techniques such as Principal Component Analysis (PCA) and t-distributed Stochastic Neighbor Embedding (t-SNE) are widely used.

#### 4.2.3. Density Estimation

Density estimation involves estimating the underlying probability distribution, p(x). Methods such as Gaussian Mixture Models (GMMs) and Kernel Density Estimation (KDE) are commonly employed.

#### 4.2.4. Association Rule Mining

Association rule mining is an unsupervised learning technique used to identify meaningful co-occurrence patterns in large datasets. A rule is expressed as A⇒B, where *A* and *B* are disjoint itemsets. The key quality measures include support (joint frequency), confidence (conditional probability), and lift (statistical dependency) [74]. Algorithms such as Apriori and FP-Growth efficiently search the rule space by pruning the low-support candidates. Because the resulting rules are inherently interpretable, this technique complements more complex machine learning models in safety-critical domains.

### 4.3. Semi-Supervised and Self-Supervised Learning

Semi-supervised learning uses a small set of labeled data ${\left\{\left(x_{i},y_{i}\right)\right\}}_{i=1}^{l}$ and a large set of unlabeled data ${\left\{x_{j}\right\}}_{j=l+1}^{l+u}$. The goal is to exploit the structure of unlabeled data to improve generalization. A common assumption is the cluster or manifold assumption, in which the decision boundaries should be located in low-density regions.

Self-supervised learning, a recent and powerful variant, constructs surrogate tasks (e.g., predicting masked inputs or temporal order) to generate supervisory signals from the unlabeled data. This has proven to be especially effective in natural language processing (e.g., BERT) and computer vision (e.g., SimCLR).

### 4.4. Reinforcement Learning

Reinforcement Learning (RL) is a learning paradigm in which an agent interacts with an environment and learns to select actions that maximize long-term rewards. At each time step, the agent observes the state of the environment, chooses an action, and receives a scalar feedback signal (reward) that evaluates the immediate consequences of that action. Through repeated interactions, the agent improves a decision-making rule, that is, a *policy*, that maps states to actions to optimize cumulative performance over time [75].

Formally, RL addresses sequential decision-making under uncertainty by modeling the environment as a Markov Decision Process (MDP), defined by the tuple (S,A,P,R,γ), where:

- S: the set of possible states;
- A: the set of possible actions;
- $P(s^{\prime }|s,a)$: the transition probability from state *s* to state $s^{\prime }$ under action *a*;
- R(s,a): the reward function associated with taking action *a* in state *s*;
- γ∈[0,1): the discount factor weighting future rewards.

The objective is to learn a policy π:S→A that maximizes the expected discounted return as follows:

(2) $$J\left(\pi \right)=E_{\pi }\;\left[\sum_{t=0}^{\infty }\gamma^{t}R\left(s_{t},a_{t}\right)\right].$$

A central concept in RL is the estimation of value functions—such as $V^{\pi }\left(s\right)$ or $Q^{\pi }\left(s,a\right)$—which quantify the expected return of states or state–action pairs under a policy. Optimal policies are characterized by Bellman optimality equations, which form the foundation of classical and modern algorithms. RL methods are generally grouped into value-based approaches (e.g., Q-learning), policy-based approaches (e.g., REINFORCE), and actor–critic methods that combine both. Recent surveys highlight how deep neural networks have enabled RL to scale to high-dimensional spaces while introducing challenges related to stability, exploration, and sample efficiency [76,77]. Common techniques for addressing these challenges include experience replay, target networks, entropy regularization, variance-reduction strategies, and improved policy optimization schemes. From a control perspective, RL is closely linked to Adaptive Dynamic Programming (ADP), where actor–critic structures approximate optimal control laws for nonlinear or partially known systems. Overall, RL provides a principled framework for learning sequential decision policies directly from interaction, balancing exploration and exploitation, and leveraging function approximation to extend decision-making capabilities to complex, uncertain environments [75,76,77,78].

### 4.5. Other Paradigms

#### 4.5.1. Symbolic and Logic-Based Learning

Symbolic learning methods, such as Inductive Logic Programming (ILP), aim to learn interpretable rules from structured data. ILP systems operate within a logical framework, learning hypotheses *H* such that

(3) B∪H⊧E

where *B* is the background knowledge, and *E* is the set of positive examples.

#### 4.5.2. Ensemble and Hybrid Methods

Ensemble methods combine multiple models to improve the predictive performance. Techniques include:

- Bagging: Reduces variance by averaging predictions over bootstrap samples.
- Boosting: Sequentially focuses on hard examples to reduce bias.
- Stacking: Learn a meta-model to combine base learners.

Formally, an ensemble predictor F(x) may be expressed as

(4) $$F\left(x\right)=\sum_{m=1}^{M}\alpha_{m}f_{m}\left(x\right)$$

where $f_{m}$ is the base learner and $\alpha_{m}$ are weights.

#### 4.5.3. Learning from Interaction and Metalevel Adaptation

Intelligent agents must learn not only from static datasets but also from interactions and experiences. The concept of metalevel learning was introduced by Russell and Norvig [73], where agents adapt their learning strategies based on the performance feedback. This includes learning to select features, tune hyperparameters, and choose among the learning algorithms.

The taxonomy of learning paradigms in artificial intelligence encompasses a wide range of approaches, each suited to different types of data and problem settings. Supervised, unsupervised, semi-supervised, and reinforcement learning form the core paradigms, whereas symbolic methods and ensemble techniques offer additional flexibility and interpretability. The integration of these paradigms, along with advances in self-supervised learning and metalevel adaptation, continues to drive progress in this field.

#### 4.5.4. Transformer-Based Architectures

Transformer-based models have recently emerged as a powerful alternative to traditional sequence and image processing architectures. Originally introduced for natural language processing [79], transformers rely on self-attention mechanisms that enable the modeling of long-range dependencies without the sequential constraints of recurrent neural networks. This capability has led to state-of-the-art performance across various domains.

In computer vision, Vision Transformers (ViTs) [80] have demonstrated competitive accuracy with convolutional networks by treating images as sequences of patches and learning global spatial relationships through attention. For time-series analysis, transformer variants such as the Informer [81] compress long input sequences and improve forecasting efficiency, which is especially relevant for railway monitoring applications involving long-term degradation patterns, sensor fusion, and anomaly detection.

Given their flexibility, scalability, and strong generalization capability, transformer-based architectures represent a promising direction for future research in railway infrastructure maintenance, particularly for tasks requiring integration of heterogeneous signals, complex temporal dynamics, or high-resolution spatial inspection.

## 5. AI Approaches Applied to Railway Infrastructure Maintenance

This section presents the main findings of the systematic review, offering an evidence-based synthesis of how Artificial Intelligence techniques have been applied to railway infrastructure maintenance. The results are organized by subsystem—track, energy, and control-command and signaling—following the classification of the European Railway Agency (ERA) and linking them to the technical areas of ADIF for practical relevance. For each category, the predominant maintenance models, types of data analyzed, and AI methods used are highlighted. Representative studies are summarized to illustrate real-world applications and performance outcomes, providing a clear picture of the current state of the art before discussing challenges and future directions in Section 6.

### 5.1. Railway Infrastructure Assets Classification

Railway infrastructure is a complex system composed of multiple interconnected subsystems, each with its own maintenance challenges. The European regulatory framework, such as *Directive (EU) 2016/797* [82] on the interoperability of the railway system and its transposition into national legislation, such as *Spain’s Law 38/2015 on the railway sector* [83], establishes a structural and functional division that serves as the basis for asset management. Table 1 illustrates the correspondence between the ADIF technical areas and the EU rail system subsystems, as established by the Technical Specifications for Interoperability (TSIs). The subsystems of interest in this study are those that genuinely reside within the physical infrastructure of the railway network: Infrastructure, Energy, and Control-Command and Signaling, along with functional aspects such as operations and maintenance.

Infrastructure maintenance encompasses a set of operations (preservation, repair, replacement, and upgrading) aimed at keeping assets in a safe and operational condition. The public company ADIF, manager of the Spanish General Interest Railway Network (RFIG), structures its maintenance procedures based on these guidelines. For example, the corrective maintenance procedure defines the phases from incident detection to resolution, distinguishing between immediate and deferred maintenance procedures. This procedure applies to technical areas such as Track Infrastructure, Safety Installations, Level Crossings, Energy, Telecommunications, and Protection and Security Installations. This last area is not considered within the scope of this study because it lacks a direct link to fixed infrastructure assets and does not belong to the essential domain of railway operations. Table 2 provides a structured classification of railway infrastructure assets by subsystem and technical area, linking them to maintenance models and their representative scientific contributions.

#### 5.1.1. Infrastructure Subsystem

This subsystem includes tracks and civil works. The track comprises rails, sleepers, fasteners, and ballast. Failures in this subsystem, such as rail defects (cracks, wear, and corrugation), track geometry degradation, and ballast deterioration, are among the leading causes of accidents and speed restrictions.

- Rail Defects: Issues such as rolling contact fatigue (RCF), including squats, which represent a characteristic manifestation of RCF, and defective welds are critical sources of deterioration. Early detection using non-destructive inspection techniques (e.g., ultrasonic testing) and data analysis via AI is essential to prevent rail fractures. Techniques such as ultrasonic and vibration analyses combined with AI models (CNN, LSTM, and SVM) have been successfully applied in the detection of rail defects [84]. In this way, it is important to note that defect detection is inherently linked to defect severity classification. Recent publications emphasize that assessing defect severity provides critical insights into prioritizing maintenance interventions. For example, Hu et al. [85] introduced a deep learning–based severity evaluation framework that classifies rail-surface deterioration from level 0 (no defect) to level 7 (severe), showing that severity grading substantially improves the interpretability and utility of automated inspection systems.
- Track Geometry: Degradation in parameters such as leveling, alignment, gauge, and cant directly affects safety and comfort. Continuous monitoring using track inspection vehicles generates large volumes of data suitable for ML-based predictive modeling. Ensemble classifiers and gamma process models have been used to predict geometry degradation [86], while GIS-integrated ML approaches [87] or Scan-to-BIM geometric localization framework [88] have been proposed for defect localization, improving scheduling and traceability.
- Turnouts: These are complex and costly components. Failures in elements such as switch blades, frogs, or actuators significantly impact network availability. Artificial intelligence-based structural health monitoring strategies using Digital Twins have been proposed to address maintenance under various conditions [89].

#### 5.1.2. Energy Subsystem

This subsystem provides traction power and includes substations, overhead contact lines (catenary), and the return circuit.

- Catenary and Pantograph: Their interaction is a common source of failure. Contact wire wear, electrical arcing, or insulator issues can cause serious disruptions. Monitoring using thermal and visual cameras, combined with ML, enables anomaly detection and failure prediction. LiDAR-based 3D imaging and AI/ML-based asset extraction have been applied to overhead catenary systems [90]. Furthermore, Wang et al. [91] developed a deep semantic model that automatically identifies defect severity levels in catenary records, underscoring how severity assessment complements detection to enable prescriptive, risk-informed maintenance planning.
- Substations: These critical installations transform and distribute energy. Predictive maintenance of components such as transformers and circuit breakers is vital to a reliable power supply. AI-based methods using FPCA and DTW have been proposed for the predictive maintenance of railway energy systems [92].

#### 5.1.3. Control-Command and Signaling Subsystem

This subsystem includes all ground and onboard equipment necessary to ensure the safety and control of the train’s circulation. In this contribution, however, the focus is exclusively on the fixed infrastructure components of these systems, leaving out onboard elements. Thus, it includes interlocking systems, track circuits, axle counters, beacons, and the European Rail Traffic Management System (ERTMS).

- Track Circuits: Essential for train detection. Failures can lead to false occupancy (“track occupied”) or, more dangerously, false clearance (“track free”), with serious safety implications. Signal analysis using ML can predict degradation. Deep learning and ensemble models have been applied to signaling systems [93].
- ERTMS: As the European standard for signaling, its implementation and maintenance are key to interoperability. ERTMS operational data analysis is an emerging field for predictive maintenance. AI-based asset management frameworks that integrate ERTMS data have been proposed [93], and predictive-cognitive maintenance strategies using Digital Twins and CPS are being explored [65].

### 5.2. Results of the Review: Applications of AI and ML

The literature review has identified a wide range of applications of Artificial Intelligence (AI) and Machine Learning (ML), most of which are focused on predictive maintenance (PdM) of railway infrastructure, with additional approaches addressing prescriptive (PsM) and preventive maintenance (PvM). As shown in Table 2, this classification provides a comprehensive mapping between railway subsystems, technical areas, and maintenance models, serving as a reference framework for the detailed analysis presented in the following subsections.

**Table 2:** Comprehensive classification of scientific contributions organized by subsystem (ERA) and technical area (ADIF), linking each category to the predominant maintenance models. Due to the interdependencies among subsystems and the methodological approaches proposed by authors, a single publication may be classified under multiple subsystems.

Asset Clasification		Maintenance Model		
Subsystem (ERA)	Technical Area (ADIF)	PvM	PdM	PsM
Infrastructure	Infrastructure and Track	Guler [94], Macedo et al. [95]	Guler [96], Yokoyama [97], Lee et al. [98], Marsh et al. [99], Cárdenas-Gallo et al. [86], D’Angelo et al. [100], Lee et al. [101], Jamshidi et al. [102], Liu et al. [103], Durazo-Cardenas et al. [104], Tam et al. [105], Gbadamosi et al. [106], Allah Bukhsh et al. [67], Ou et al. [107], Lasisi and Attoh-Okine [108], Lopes Gerum et al. [109], Yao et al. [110], Lu et al. [111], Zhang et al. [112], Chen et al. [113], Consilvio et al. [114], Shubinsky et al. [115], Ghofrani et al. [116], Stypułkowski et al. [117], Zhang et al. [118], Daniyan et al. [119], Dirnfeld et al. [13], Popov et al. [120], Vale and Simões [121], Mohammadi and He [122], Nampalli [123], Nagy et al. [124], Di Costanzo et al. [93], Kumari et al. [125], Guillén et al. [126], Ariyachandra et al. [65], Bianchi et al. [89], Nwamekwe et al. [127], MajidiParast et al. [128]	Durazo-Cardenas et al. [104], Oneto et al. [129], MajidiParast et al. [128], MajidiParast et al. [128]
Energy	Energy		Takikawa [130], Liu et al. [103], Lin et al. [131], Wang et al. [66], Liu et al. [132], Karaduman and Akin [133], Patwardhan et al. [90], Ariyachandra et al. [65]	Wang et al. [66]
Control- Command and Signaling (Trackside)	Safety Installations		Yokoyama [97], Takikawa [130], de Bruin et al. [134], Durazo-Cardenas et al. [104], Hu et al. [135], Gao et al. [136], Arslan and Tiryaki [137], Chen et al. [113], Consilvio et al. [114], Gbadamosi et al. [106], Soares et al. [138], Nampalli [123], Kumari et al. [125], Guillén et al. [126], Ariyachandra et al. [65], Nwamekwe et al. [127]	Durazo-Cardenas et al. [104], Oneto et al. [129]
	Telecommunications		Hu et al. [135], Gao et al. [136], Kalapati et al. [92]	

#### 5.2.1. Contributions Related to Infrastructure Subsystem (Track)

The infrastructure subsystem, particularly the track, focuses the largest number of investigations due to its criticality and the abundance of monitoring data available. One of the most studied areas is the detection and prediction of rail and weld defects. Early detection of rail defects, such as cracks or squats, is crucial to prevent rail breakage, one of the main causes of derailments. Deep learning techniques, especially Convolutional Neural Networks (CNNs), have shown great effectiveness in analyzing rail surface images. For example, Lu et al. proposed SCueU-Net, a segmentation model combining U-Net and saliency cues, with 99. 76% precision in damage detection [111]. Similarly, Zhuang et al. developed a data-driven double-layer framework for automated crack inspection using extended Haar-like features and ensemble classifiers [139].

In addition to image-based approaches, sensor signal-based methods are widely used. Monitoring using Axel Box Accelerators (ABAs) is a common technique to detect irregularities. Jamshidi et al. proposed a decision support system combining ABA data and video analysis, using a Fuzzy Inference System and MILP optimization to plan rail grinding [102]. Welds, being weak points on the track, are also a focus of predictive modeling. Yao et al. developed a model using Extreme Learning Machine (ELM), Random Forest, and SVM to predict weld defects, significantly reducing the inspection workload while maintaining safety standards [110].

Another key area is the prediction of track geometry degradation, which is essential for planning maintenance operations such as tamping. Artificial Neural Networks (ANNs) have been used effectively in this context. Guler applied ANNs to predict geometry deterioration rates using historical measurement and intervention data [96]. Popov et al. used ANNs to classify track segments by quality and assess maintenance efficiency [120]. To improve robustness, ensemble models have also been explored. Cárdenas-Gallo et al. proposed a classifier combining the Gamma Process, Logistic Regression, and SVM to predict the evolution of geometry defects [86]. Lasisi and Attoh-Okine used multilayer stacking to combine learners for the prediction of fatigue defects [108], while Mohammadi et al. applied XGBoost optimized by Bayesian methods to model geometry degradation at fine resolution [140].

Turnouts (switches) are another critical and complex component of the infrastructure subsystem. Failures in point machines can cause severe disruptions. Several studies have focused on the analysis of electrical and acoustic signals to detect faults. Ou et al. proposed a method using PCA/LDA and a balanced SVM for fault diagnosis based on monitoring data, achieving 99% precision [107]. Arslan and Tiryaki compared ANN and SVM to predict point machine failures, finding ANN to be more accurate [137]. Lee et al. used MFCC and SVM for acoustic fault detection, achieving a precision of over 94% [98]. Additionally, Chen et al. developed a method combining Auto-Associative Kernel Regression (AAKR) and Genetic Programming (GP) to estimate the Remaining Useful Life (RUL) of turnout, providing an explicit relationship between health indicators and lifespan [113]. Table 3 summarizes representative AI-based contributions for the infrastructure subsystem, detailing the type of data analyzed, evaluation metrics, and reported performance for predictive maintenance approaches.

#### 5.2.2. Contributions Related to the Energy Subsystem

Research within this subsystem primarily targets the pantograph–catenary interface, a critical element for reliable electric train operation. Advances in overhead line equipment (OLE), pantographs, and traction power assets have driven the development of predictive maintenance strategies and, to a lesser extent, prescriptive approaches, leveraging a range of AI-based techniques.

For the pantograph–catenary interface, Karaduman and Akin [133] propose an IoT-based framework that combines image correlation and temperature detection with a Mamdani fuzzy classifier, achieving high precision in categorizing pantograph health states and allowing condition-based interventions. Detection of anomalies in the pantograph-catenary interaction is essential to prevent service interruptions. Karakose and Yaman proposed a thermal imaging system combined with a Complex Fuzzy System adapted to seasonal conditions to detect anomalies in the pantograph contact area [141]. Complementarily, Patwardhan et al. [90] introduce a LiDAR-driven architecture for OLE monitoring, employing point cloud segmentation, clustering, and AI-based classification within a microservices pipeline. This approach supports Digital Twin creation and integrates VR/AR interfaces for anomaly visualization, facilitating data-to-decision workflows for large-scale predictive maintenance.

Environmental risk modeling is addressed by Lin et al. [131], who apply AdaBoost ensemble learning to historical fault and meteorological data, establishing strong correlations between weather conditions and catenary failures. Their model attains approximately 89% accuracy, enabling proactive scheduling under adverse climatic scenarios.

For traction power equipment, Wang et al. [66] combine a data-driven LSTM recurrent neural network with a physics-informed sample generator to predict optimal maintenance windows for gas-insulated switchgear. This hybrid paradigm mitigates data scarcity and improves prognostic reliability. At the system level, Liu et al. [132] propose a hierarchical fault detection and isolation scheme for hybrid AC/DC grids, using Gated Recurrent Unit (GRU) networks deployed on FPGA hardware. The architecture achieves sub-millisecond latency and over 93% classification accuracy in 30 fault scenarios, ensuring real-time resilience in complex electrification environments. Table 4 provides a comparative summary of notable AI-based contributions for the energy subsystem, highlighting monitored assets such as pantograph–catenary systems and traction power equipment, the types of data used (thermal images, meteorological variables, electrical signals), and key performance indicators including accuracy and latency.

#### 5.2.3. Contributions Related to the Control-Command and Signaling Subsystem

This is an area of growing interest, as failures in signaling systems have a direct and severe impact on both safety and network capacity. One of the key components in this subsystem is the track circuit, which is essential for train detection. Hu et al. [135] proposed a fault prediction method based on grey theory and expert systems, using a dynamic model to improve prediction accuracy. Recurrent Neural Networks (RNNs), specifically LSTM architectures, are employed to diagnose faults in track circuits, demonstrating the ability of these models to learn from temporal data sequences and achieve high classification accuracy [134].

The rise in prescriptive maintenance also finds relevance within railway engineering. Although contributions in this specific area remain relatively scarce, one notable work is that of Consilvio et al. [142], which presents an application designed to proactively address failures that lead to false occupancy detection. Such failures present a significant challenge to railway operations, directly affecting both infrastructure capacity and service reliability. This contribution goes beyond traditional predictive maintenance (PdM) by introducing an advanced decision-making layer, where maintenance strategies are guided or fully automated through a machine learning-driven approach. However, it does not explicitly address the role of neural networks in this context.

Recent advances expand this scope through the integration of IoT and AI for CCS assets, enabling real-time monitoring of interlockings and track circuits, remote inspections, and predictive workflows within common data environments [106]. In addition to this systemic view, Gao et al. [136] address the physical integrity of the track equipment with GPS-corrected image matching, reducing false positives in anomaly detection and ensuring precise localization of defects.

For turnout systems, which are critical to route setting, Soares et al. [138] employs unsupervised learning to classify operational states without labeled data, while Chen et al. [113] advances RUL estimation through feature fusion and Genetic Programming, offering interpretable models that overcome the opacity of black-box approaches. At a higher level of autonomy, Kumari et al. [125] demonstrates how AI-driven predictive maintenance frameworks can reduce costs and downtime by integrating sensor analytics with adaptive scheduling.

Another critical area is radio communications, which support signaling and control operations. Kalapati et al. described an AI-based method for the predictive maintenance of railway radio communication systems, using Functional Principal Component Analysis (FPCA) to define health indicators and supervised ML classifiers to detect specific degradations in GSM-R and related networks [92]. Table 5 summarizes selected contributions for CCS assets, including track circuits, turnout systems, and general signaling components, specifying the data types analyzed (temporal signals, force/power signals, sensor data) and the reported performance metrics such as accuracy, RMSE, and cost reduction.

#### 5.2.4. Holistic Approaches and Integrated Platforms

Beyond individual components, several studies propose integrated frameworks that encompass multiple aspects of railway maintenance, often incorporating concepts such as Digital Twins and Cyber–Physical Systems (CPSs). Gbadamosi et al. proposed an IoT-enabled strategy for real-time monitoring and predictive maintenance of railway assets, identifying priority areas such as real-time condition monitoring and decision support [106]. Similarly, Zhang et al. designed a predictive maintenance platform architecture based on IoT and AI, covering asset monitoring, cloud-based data analysis, and decision support [118].

Digital Twins and CPS represent the frontier of asset management. A Digital Twin is a virtual replica of a physical asset, updated in real-time via sensor data. Ariyachandra et al. proposed a conceptual framework that integrates Digital Twins and CPS to improve predictive maintenance, enabling automated decision-making through seamless data exchange [65]. Liu et al. presented a CPS architecture enabled by industrial AI for Prognostics and Health Management (PHM) in high-speed railway systems, creating cyber twins of key subsystems to improve transparency and decision efficiency [103]. Integration of ML with Digital Twins allows simulations and “what-if” scenarios that optimize maintenance strategies proactively. The recent literature further reinforces the transformative potential of Digital Twin-based platforms.

These architectures are increasingly used to enhance the spatial and semantic characterization of defects. The DT framework discussed by Futai et al. [143] illustrates how multi-modal sensing, simulation, and data fusion enable refined condition assessments. More recent developments, such as the DefectTwin approach [144], combine DTs with multimodal AI and Large Language Models to improve defect interpretation—including severity evaluation and spatial mapping—while reducing data requirements for model training and deployment.

Despite their transformative potential, the deployment of integrated platforms based on Digital Twins and CPS requires overcoming significant challenges in data governance, system interoperability, and regulatory compliance. Table 6 compiles these contributions into a unified framework, linking maintenance models with the AI techniques implemented, and highlighting the evolution from predictive to prescriptive approaches, as discussed in the following section.

## 6. Discussion of Challenges, Implications, and Future Trajectories

### 6.1. The Foundational Challenge: Data Quality, Integration, and Accessibility

A recurring and central theme across nearly all studies is the critical dependency on high-quality, accessible, integrated data. This fragmentation is recognized globally; railway companies surveyed by UIC - Rail System Department and McKinsey & Company [18] indicated that limited data availability and quality, including siloed data infrastructure, remain key challenges for building solutions at scale. The promise of big data analytics to revolutionize maintenance can only be realized if the underlying data are sound. However, the reality within many railway organizations have fragmented information landscapes. Data often resides in isolated silos, managed by different departments, and stored in disparate, nonintegrated databases. This creates a significant barrier to obtaining a holistic view of asset health that is necessary for effective PdM.

Crucial information is distributed across multiple databases, with much of the most detailed inspection data held on paper forms, hindering the ability to link asset characteristics with fault history. This fragmentation is a major obstacle identified by numerous researchers; ref. [130] emphasizes that JR East’s divisional systems are independent and lack a common data strategy, making a unified platform essential for “Smart Maintenance”. Similarly, the DAYDREAMS project [129] aims to address this issue by integrating data for prescriptive analytics. The need for a Common Data Environment (CDE) is further stressed by Gbadamosi et al. [106], who argue that it would break down the current discipline-based data silos and enable IoT-based predictive systems.

The quality of the data presents another major hurdle. Datasets are often noisy, incomplete, or have inconsistent asset identifications. A critical issue for supervised learning models is the problem of imbalanced data; hazardous failures are, by definition, rare events, which means that there is a scarcity of failure data available for training models to predict them. Shubinsky et al. [115] address this challenge in their work on predicting hazardous track failures, while Ou et al. [107] propose a modified Support Vector Machine (SVM) to handle the imbalance between rare fault samples and abundant normal samples in turnout systems. Mohammadi and He [122] apply the Adaptive Synthetic Sampling Approach (ADASYN) to overcome data imbalance when predicting rail defects from track geometry data. Wang et al. [66] go further by developing sample generators based on physical degradation models to create synthetic data for training their LSTM-RNN predictor for power equipment.

This challenge is not merely technical but deeply organizational. The success of any digital transformation requires a fundamental rethinking of the data strategy and governance. Patwardhan et al. [90] propose a cloud-based microservices architecture for processing 3D imaging data from catenaries, exemplifying the kind of structured approach needed to effectively manage data extraction, integration, and analysis. Le-Nguyen et al. [148] emphasize the difficulties of working with data streams that have no predefined format, requiring sophisticated methods like their InterCE framework just to extract meaningful operational cycles before any analysis can begin.

### 6.2. The Interpretability Dilemma: Bridging the Gap Between “Black Boxes” and Decision-Makers

As the sophistication of machine learning models increases, so does their complexity, often turning them into “black boxes” whose decision-making processes are opaque to human users. This issue is structural: AI technologies, particularly complex ones such as Deep Learning, solve problems but remain rather opaque [5]. This opacity creates fundamental challenges in safety-critical contexts, leading to ethical concerns. Specifically, AI systems can perpetuate or amplify existing biases if the training data are not representative, potentially leading to inaccurate maintenance decisions or misclassification of critical faults in safety-critical railway systems [19]. This lack of interpretability is a major barrier to adoption, particularly in a safety-critical industry such as railways, where engineers and managers need to trust and understand the reasoning behind a model’s prediction before acting on it.

Although deep learning models, such as Convolutional Neural Networks (CNNs), show great promise for tasks such as image-based defect recognition, their opacity can be a significant drawback [137]. In response, there has been a growing emphasis on explainable AI (XAI). Allah Bukhsh et al. [67] employ the LIME (Local Interpretable Model-Agnostic Explanations) framework to provide instance-level explanations for their tree-based models that predict the maintenance needs of switches. This allows domain experts to understand which specific features contribute to the prediction of a model for a single maintenance trigger, thus fostering trust and enabling model improvement.

Jamshidi et al. [102] uses a Mamdani fuzzy inference system precisely because of its interpretability, allowing expert knowledge to be systematically encoded into readable rules. Hu et al. [135] combines grey theory with an expert system for the prediction of track circuit faults using simple and clear production rules. Arslan and Tiryaki [137] find that while Artificial Neural Networks (ANNs) yield higher predictive accuracy, their adoption can be stalled by a lack of trust. In contrast, simpler models, such as logistic regression used by Vale and Simões [121], offer greater transparency. Therefore, future research must focus not only on improving predictive power but also on developing robust XAI techniques that can make these powerful tools accountable to human decision-makers.

### 6.3. Technological Integration and the Rise of Holistic Systems

The most advanced research points toward a future where maintenance is not managed by isolated predictive models but by integrating holistic systems that provide a comprehensive view of the entire railway network. The concepts of Digital Twins (DTs) and Cyber–Physical Systems (CPSs) are central to this vision. A DT, as a dynamic virtual replica of a physical asset, offers a platform to fuse data from multiple sources (e.g., IoT sensors, inspection data, and operational schedules) and simulate asset behavior under various conditions.

Several studies have underscored this trend. Sresakoolchai and Kaewunruen [146] propose integrating Deep Reinforcement Learning with a DT to improve maintenance efficiency, using the DT as a data management platform for long-term health tracking. Similarly, Ariyachandra et al. [65] proposed a DT-CPS framework for an interconnected ecosystem that enables access to data in near real-time. The development of a “Cyber Twin” for high-speed rail systems, as described by Liu et al. [132], emphasizes the creation of virtual models of key components to improve condition transparency and decision-making efficiency. This trend is also highlighted by Nwamekwe et al. [127], who reviewed how Machine Learning-Augmented Digital Twin Systems (ML-DTS) are transforming predictive maintenance. The practical implementation of such systems was explored by Guillén et al. [126] through the design of sensorized rail pads with embedded piezoelectric sensors, which provide the real-time data necessary to feed these digital models.

This move toward integrated systems also implies a shift from predictive to prescriptive maintenance. While PdM predicts what will happen, PsM recommends what should be done to prevent failure. The DAYDREAMS project, as described by Oneto et al. [129], explicitly focuses on developing AI-based prescriptive analytics. The autonomous system proposed by Durazo-Cardenas et al. [104] embodies this prescriptive ideal by combining condition data with planning and cost models to automatically schedule interventions. A novel approach was presented by MajidiParast et al. [128], who used Graph Convolutional Networks (GCNs) to model the spatial interdependencies of the railway network, allowing for optimal intelligent predictive maintenance that considers the network as a whole rather than isolated segments. As summarized in Table 6, the reviewed literature spans a wide range of AI paradigms, from classical machine learning to deep learning and reinforcement learning, reflecting the heterogeneity of methods required to address different asset types and maintenance objectives.

However, the path to fully realizing DTs and PsM systems is steep. This requires solving the aforementioned data integration challenges, ensuring interoperability between systems, and managing high implementation costs. Moreover, it requires a significant organizational shift, as highlighted by the IoT implementation strategy proposed by Gbadamosi et al. [106], which calls for moving away from siloed departments toward a culture of cross-disciplinary data sharing and collaboration. UIC reported six key components necessary for the successful implementation of AI on a large scale [18]: defining a strategic roadmap, securing AI-specific roles and skills, adopting an agile operating model, setting up the right technology and data management, and ensuring effective adoption and scaling. This strategic framework confirms that integration is not purely technological but requires strong organizational transformation.

### 6.4. The Diversity of Methods and Applications

The breadth of research indicates that no single technology or method is a panacea for all problems. The choice of technique is highly dependent on the specific asset and nature of the available data. As consolidated in Table 6, the reviewed literature spans a wide range of AI paradigms and maintenance models, reflecting the heterogeneity of the approaches required for different subsystems. These tables collectively illustrate how the data types, evaluation metrics, and AI techniques vary significantly across the infrastructure, energy, and control-command domains. In addition to serving as a descriptive synthesis, these comparative summaries provide a practical foundation for decision-making in railway asset management. By mapping AI methods to specific maintenance models and subsystems, infrastructure managers and researchers can identify the most suitable techniques for given operational contexts. Furthermore, this structured classification supports future standardization efforts by highlighting recurring patterns and gaps that can inform the development of interoperable frameworks and guidelines for AI-driven maintenance in the railway sector.

A wide range of methods has been applied for track geometry. Guler [96] used Artificial Neural Networks (ANNs) to predict the deterioration of the track geometry for the Turkish state railways. Lee et al. [101] also used ANN, in conjunction with Support Vector Regression (SVR), to predict the Track Quality Index (TQI) in Korea, while Popov et al. [120] used a single-hidden-layer ANN to classify the condition of track segments after tamping. Cárdenas-Gallo et al. [86] developed an ensemble classifier that combined a gamma process, logistic regression, and SVMs to predict degradation. Bergquist and Söderholm [145] proposed a control chart approach for assessing linear assets such as tracks, demonstrating earlier problem detection than traditional safety limits. More recently, Nagy et al. [124] compared linear, exponential, and ANN models for the same purpose in Hungary. Finally, a study by Giunta and Leonardi [87] proposed a data-driven methodology to locate isolated geometric defects to support preventive maintenance strategies.

Computer vision and image processing are prevalent for rail defects. Lu et al. [111] proposed SCueU-Net for efficient damage detection on rails, and Sysyn et al. [149] used image processing and ML methods to predict fatigue from rail contact at crossings. Physics-informed models are also gaining traction; Ghofrani et al. [116] integrated Finite Element modeling with a Bayesian framework to predict the arrival rate of rail breaks. Lasisi and Attoh-Okine [108] proposed a multilayer stacking ensemble of machine learning models to improve the prognosis of rail defects, compensating for the shortcomings of the classical Weibull analysis. The integration of AI and IoT for predictive operations was highlighted by Kumar [84], who developed a framework for a Rail Defect Measurement System (RDMS) using smart sensors and machine learning algorithms for real-time anomaly detection.

Various machine learning techniques are used for turnouts (switches). Soares et al. [138] applied unsupervised techniques, such as k-means, to prevent faults on switch machines, while Allah Bukhsh et al. [67] used tree-based classification models for predictive maintenance. Sound analysis is another innovative approach, as demonstrated by Shafique et al. [150], who used Mel-Frequency Cepstrum Coefficients (MFCCs) and SVMs to detect and diagnose faults from audio data.

In power systems, the focus is often on specific components of the system. Karakose and Yaman [141] developed a complex fuzzy system-based thermography approach for predictive maintenance on pantograph-catenary systems. Liu et al. [132] designed a real-time hierarchical neural network using Gated Recurrent Units (GRUs) for fault detection in hybrid AC/DC grids. Lin et al. cite Lin2019AConditions developed a fault prediction method for catenary systems based on meteorological conditions using the AdaBoost algorithm. Kalapati et al. [92] proposed an AI-based method using Dynamic Time Warping (DTW) and Functional Principal Component Analysis (FPCA) to monitor the health of train-to-ground radio communications.

For other infrastructure components, Stypułkowski et al. [117] presented a concept for using thermographic imaging to detect failures in electric heating devices for turnouts (EORs), supported by machine learning for automatic thermogram analysis. A study by D’Angelo et al. [100] evaluated the potential advantages of bitumen-stabilized ballast (BSB) through an integrated model that estimates maintenance interventions throughout the life cycle. For insulated rail joints (IRJs), Bianchi et al. [89] combined a Digital Twin with AI-based classifiers to predict the structural health based on the preload conditions of the bolt.

Maintenance optimization is a significant field. Macedo et al. [95] proposed a Mixed Integer Programming (MIP) formulation and a Variable Neighborhood Search (VNS) algorithm for scheduling preventive maintenance with resource constraints. Guler [94] used genetic algorithms to optimize track maintenance and renewal work plans. Mohammadi and He [122] applied a Double Deep Q-Network (DDQN) for optimal rail renewal and maintenance planning, framing the problem within a Deep Reinforcement Learning (DRL) context. This diversity is also reflected in the variety of data sources used, from traditional track geometry car measurements to novel sources.

### 6.5. Implications for Practice and Future Directions

The synthesized research provides a clear roadmap for infrastructure management. The immediate implication is the need for a robust data strategy to address these challenges. Without a concerted effort to break down data silos, standardize data formats, and ensure data quality, the potential of advanced analytics remains largely untapped. The adoption of a CDE, as advocated by Gbadamosi et al. [106], appears to be a necessary and logical first step in this direction. Furthermore, research indicates that practical value can be derived even without fully autonomous systems. Decision Support Systems (DSSs), which leverage predictive models to provide insights to human experts, offer a pragmatic intermediate step. Models based on interpretable techniques, such as decision trees or fuzzy logic, can empower maintenance engineers by providing data-backed recommendations that they can evaluate based on their domain expertise.

In addition to technical advancements, regulatory preparation is paramount. The European Union has established a comprehensive risk-based framework for deploying AI systems. A long-standing principle in the railway sector dictates that authorization to place AI on the market—particularly for safety-critical applications—will be granted only if the human–machine system as a whole is considered [5]. This regulatory focus ensures that providers comply with stringent requirements regarding high-quality data, risk management systems, technical documentation, and permanent human oversight [19]. These elements underscore the strategic importance of accountability and transparency in future deployment.

Another key dimension is sustainability, where AI-enabled predictive maintenance plays an increasingly strategic role. The recent scientific literature highlights that advanced AI methods can significantly improve the environmental performance of railway systems. In particular, Phusakulkajorn et al. [16] demonstrated that AI-driven maintenance reduces unnecessary interventions, minimizes lifecycle material consumption, lowers CO_2_ emissions, and optimizes asset utilization, thus directly contributing to sustainable railway management. Complementarily, Sarp et al. [151] show that integrating AI-powered services, IoT infrastructures, and Digital Twin ecosystems enables more energy-efficient, resource-aware, and future-proof railway networks aligned with Industry 5.0 and circular-economy principles. Together, these findings affirm that sustainability is not only an outcome of predictive maintenance but also a central driver for its adoption in modern railway infrastructure.

Looking ahead, the field is advancing on multiple fronts. The use of more sophisticated AI techniques, such as graph convolutional networks (GCNs), is emerging to model the spatial interdependencies of railway networks, recognizing that the health of a track segment is influenced by its neighbors, as proposed by MajidiParast et al. [128]. Deep Reinforcement Learning is proving to be a powerful tool for optimizing long-term maintenance and renewal plans under uncertainty, moving beyond static optimization to dynamic and adaptive policies [122]. Currently, advances in sensor technology, such as the sensorized rail pads developed by Guillén et al. [126], and the proliferation of IoT devices, will provide richer real-time data streams to feed these advanced models.

Another promising avenue for future research is the intelligent maintenance of railway telecommunications systems, which are increasingly critical for signaling, control, and operational interoperability. Despite their strategic importance, these systems (GSM-R, LTE-R, FRMCS, and IP/MPLS networks) remain underrepresented in the AI-driven maintenance literature. The integration of predictive and prescriptive models for telecom assets, leveraging real-time traffic data, signal quality metrics, and fault logs, could enable the early detection of network degradation and optimize resource allocation. Recent contributions have demonstrated the feasibility of applying AI techniques, such as Functional Principal Component Analysis (FPCA), Dynamic Time Warping (DTW), and supervised ensemble classifiers, to radio communication systems [92]. Moreover, the development of Digital Twins for telecom networks, combined with cyber–physical systems and Explainable AI, may facilitate scenario-based simulations and transparent decision-making in safety-critical environments [65,127,129].

In parallel, the emergence of 6G-enabled smart railway architectures has introduced new opportunities for integrating Reconfigurable Intelligent Surfaces (RISs), mmWave communications, and software-defined networking (SDN)-based routing optimization. These technologies offer enhanced coverage, ultralow latency, and dynamic adaptability, particularly in high-speed and NLoS scenarios [152,153]. The use of dual-coverage RIS panels and SDN-integrated ML routing frameworks [154] can be extended to predictive maintenance and fault-tolerant telecom operations, especially when combined with federated learning and edge intelligence.

In conclusion, the journey toward intelligent railway maintenance is not merely a technological challenge but a strategic one. It requires a holistic vision that integrates data, advanced analytics, and organizational processes. Although significant hurdles remain, the convergence of AI, IoT, and Digital Twin technologies offers an unprecedented opportunity to create a more resilient, efficient, and sustainable railway network for the future. Research clearly indicates that the path forward lies in building systems that are not only predictive but also prescriptive, transparent, and seamlessly integrated into the fabric of railway operations.

## 7. Conclusions

This survey provides a comprehensive overview of Artificial Intelligence (AI) applications for the maintenance of railway infrastructure from a sensing- and data-driven perspective. The reviewed literature demonstrates a rapid expansion of AI-based approaches, largely enabled by advances in sensing technologies, data acquisition systems, and signal processing methods. AI-driven solutions are increasingly influencing asset management practices in multiple rail subsystems. In this context, the railway subsystems have been systematically classified and conceptually interconnected in a didactic manner, making a relevant contribution to this work.

The analysis indicates that the track subsystem remains the domain that has been the most extensively investigated, mainly due to the availability of various sensing modalities, including vision-based systems, inertial sensors, acoustic sensing, and distributed fiber-optic sensors. Advanced AI techniques—such as Convolutional and Recurrent Neural Networks, ensemble learning methods (e.g., Random Forest and Gradient Boosting), and hybrid models—have demonstrated high performance in defect detection, condition assessment, and degradation prediction. Increasing research activity is also observed in the energy and Control, Command, and Signaling (CCS) subsystems, supported by the growing deployment of sensor networks and the availability of high-resolution visual and time-series data. Multi-sensor data fusion and heterogeneous data integration are recognized as key enablers for improving robustness, reliability, and generalization performance.

Despite these advances, several challenges continue to limit the effective deployment of AI-based maintenance solutions. Data fragmentation, heterogeneous sensor configurations, inconsistent data quality, and limited interoperability between sensing platforms hinder the development of holistic and scalable predictive systems. Therefore, the need for standardized sensing architectures, harmonized data formats, and robust data governance frameworks is evident. Furthermore, the limited interpretability of complex AI models remains a significant concern, particularly in safety-critical railway applications, where sensor reliability, transparency, and traceability of decisions are essential requirements.

Looking ahead, the field is evolving toward more integrated, autonomous, and prescriptive maintenance frameworks. Digital Twins enabled by AI and Cyber–Physical Systems (CPSs), tightly coupled with real-time sensor data streams, are expected to play a central role in enabling continuous monitoring, real-time condition assessment, and decision support. The transition from predictive to prescriptive maintenance will require not only advances in AI algorithms but also improvements in sensor integration, data fusion strategies, and closed-loop feedback mechanisms. Emerging research areas, including reinforcement learning, federated learning, and explainable AI, are expected to further enhance the reliability and trustworthiness of sensor-driven maintenance systems.

Future research should prioritize intelligent maintenance strategies for railway telecommunications infrastructure, which underpins the safe operation of sensor networks and automated Control, Command, and Signaling systems. Advanced AI techniques—such as predictive modeling of radio-link degradation and sensor-aware bandwidth optimization—can significantly enhance the reliability and availability of data transmission. The integration of Digital Twin frameworks with CPS architectures enables holistic management of both physical assets and sensing and communication infrastructures, while the adoption of FRMCS and emerging 6G technologies further improves scalability and adaptability. In addition, experimental studies on Reconfigurable Intelligent Surfaces (RISs) and machine-learning-enhanced MPLS/IP architectures demonstrate promising capabilities to improve data delivery detection, real-time diagnostics, and fault-tolerant telecommunications in next-generation railway environments.

In summary, AI is enabling a new generation of sensor-driven railway maintenance systems characterized by improved situational awareness, increased reliability, and increased operational efficiency. Realizing this vision will depend on continued research in sensing technologies, data fusion, and explainable AI, as well as close collaboration between academia and industry to address the remaining challenges and fully exploit the potential of AI-enabled sensing solutions for the maintenance of railway infrastructure.

## Figures and Tables

**Table 1:** Mapping between ADIF technical areas and EU rail system subsystems/TSIs, with brief descriptions.

ADIF Technical Area	EU Rail System		Description
	Subsystem	Legal Name of TSI	
Infrastructure and Track	Infrastructure	Commission Regulation (EU) No 1299/2014 (last modified by 2023/1694) Tracks, switches, level crossings, bridges, tunnels, station elements, safety, and accessibility equipment.	Tracks, switches, level crossings, bridges, tunnels, structural and geometric safety.
Energy	Energy	Commission Regulation (EU) No 1301/2014 (last modified by 2023/1694)	Electrification system, overhead lines, and electricity consumption measurement.
Safety Installations			
Level Crossings (control)	Control-Command and Signaling (Trackside)	Commission Regulation (EU) No 2023/1695	Equipment on the track to ensure the safety and control of train movements, including interlockings, train detection, ETCS trackside equipment, and radio communication interfaces with onboard systems.
Telecomm.			

**Table 3:** Comparative summary of notable AI-based contributions applied to the railway infrastructure subsystem, highlighting the type of data analyzed, evaluation metrics used, and reported performance results for predictive maintenance approaches.

Contribution	Asset	Data Type	Metric	Result
Lu et al. [111]	Rail Surface	Image-based	Precision	99.76%
Zhuang et al. [139]	Rail Surface	Image-based	Precision	>95%
Yao et al. [110]	Welds	Geometric Parameters	Accuracy	92%
Ou et al. [107]	Turnouts	Electrical Signals	Accuracy	99%
Arslan and Tiryaki [137]	Turnouts	Acoustic Signals	Accuracy	ANN > SVM (>94%)
Chen et al. [113]	Turnouts	Mixed Features	RUL Estim.	Error < 10%
Lasisi and Attoh-Okine [108]	Track	Track Geometry	AUC	0.93
Mohammadi and He [122]	Track	Track Geometry	RMSE	<0.05

**Table 4:** Comparative summary of notable AI-based contributions applied to the railway energy subsystem, highlighting the asset monitored, type of data analyzed, evaluation metrics used, and reported performance results for predictive maintenance approaches.

Contribution	Asset	Data Type	Metric	Result
Karaduman and Akin [133]	Pantograph– Catenary	Images + temperature (IoT)	Accuracy; Sensitivity	Accuracy ≈ 0.939; Sensitivity ≈ 0.968
Lin et al. [131]	Catenary (HSR)	Meteorological variables + fault logs	Accuracy	≈88.89%
Wang et al. [66]	Power equipment	Electrical signals + simulated/field data	Mean loss (log–cosh)	≈$4.5\times 10^{-2}$ (CV); ≈$6.1\times 10^{-2}$ (field)
Liu et al. [132]	Hybrid AC/DC traction grid	Voltages, currents, speeds, torques	Accuracy; Latency	>93%; <1 ms system evaluation

**Table 5:** AI-based approaches for Control-Command and Signaling assets, data types, and key performance metrics.

Contribution	Asset	Data Type	Metric	Result
de Bruin et al. [134]	Track Circuits	Temporal Signals	Accuracy	>90%
Chen et al. [113]	Turnout Systems	Force/Power Signals	RMSE; R^2^	5.65; 0.94
Kumari et al. [125]	CCS (General)	Sensor Data	Accuracy; Cost	96% accuracy; 25% cost reduction

**Table 6:** General compendium of relevant contributions in AI-driven railway maintenance. Classification by maintenance model and key AI methods or techniques implemented.

Ref.	Title	Methods or Techniques	Maint. Model
[96]	Prediction of railway track geometry deterioration using artificial neural networks: a case study for Turkish state railways	ANN	PdM
[145]	Data Analysis for Condition-Based Railway Infrastructure Maintenance	Statistical Process Control, Control Charts, Time Series Analysis	PdM (CBM)
[97]	Innovative changes for maintenance of railway by using ICT—To Achieve “Smart Maintenance”	ICT, CBM, Asset Management, AI-based Decision Support, Integrated Databases	PdM
[94]	optimization of Railway Track Maintenance and Renewal Works by Genetic Algorithms	Genetic Algorithms, Decision Support Systems, Expert Systems	PvM, R2F
[98]	Fault detection and diagnosis of railway point machines by sound analysis	MFCC, SVM, Audio Analysis	PdM
[99]	Using operational data for decision making: A feasibility study in rail maintenance	Bayesian Networks, Expert Systems, Decision Support Architecture	PdM
[130]	Innovation in Railway Maintenance utilizing ICT (Smart Maintenance Initiative)	IoT, Big Data Analytics, AI, CBM, Asset Management, Integrated Databases	PdM (CBM)
[95]	Scheduling preventive railway maintenance activities with resource constraints	Mixed Integer Programming (MIP), Resource Allocation, Scheduling optimization	PvM
[134]	Railway Track Circuit Fault Diagnosis Using Recurrent Neural Networks	LSTM, t-SNE, comparison with CNN	PdM
[86]	An ensemble classifier to predict track geometry degradation	Gamma Process, Logistic Regression, SVM, Ensemble Learning	PdM
[100]	Novel performance-based technique for predicting maintenance strategy of bitumen stabilized ballast	Performance-Based Evaluation, Life Cycle Assessment	PdM
[101]	Prediction of track deterioration using maintenance data and machine learning schemes	ANN, SVR, Decision Support System	PdM
[102]	A decision support approach for condition-based maintenance of rails based on big data analysis	DCNN, Fuzzy Inference System, MILP optimization, Axle Box Acceleration (ABA), Rail Video Analysis	PdM (CBM)
[104]	An autonomous system for maintenance scheduling data-rich complex infrastructure	Data Fusion, Genetic Algorithms, Heuristics, Cost modeling, Systems Engineering	PsM, PdM (autonomous CBM)
[103]	Industrial AI Enabled Prognostics for High-speed Railway Systems	Cyber–Physical Systems, AI, Edge Computing, DL, SOM, NSI	PdM
[105]	Intelligent Optical Fibre Sensing Networks Facilitate Shift to Predictive Maintenance in Railway Systems	FBG Sensors, ML, THI Index	PdM
[106]	IoT for predictive assets monitoring and maintenance: An implementation strategy for the UK rail industry	IoT, Cloud Computing, Predictive Analytics	PdM
[67]	Predictive maintenance using tree-based classification techniques: A case of railway switches	Decision Trees, Random Forest, Gradient Boosting	PdM
[140]	Exploring the impact of foot-by-foot track geometry on the occurrence of rail defects	Track Geometry Analysis, Statistical modeling, Regression	PdM
[135]	Track circuit fault prediction method based on grey theory and expert system	Grey Theory, Dynamic GM, Expert System, Fuzzy Neural Networks	PdM
[107]	A data-driven fault diagnosis method for railway turnouts	Feature extraction, PCA, LDA, Balanced SVM, MMS data	PdM
[108]	Machine Learning Ensembles and Rail Defects Prediction: Multilayer Stacking Methodology	Ensemble Learning, GBM, SVM, Logistic Regression	PdM
[131]	A fault prediction method for catenary of high-speed rails based on meteorological conditions	AdaBoost, Decision Trees	PdM
[109]	Data-driven predictive maintenance scheduling policies for railways	Random Forests, RNN, Markov Decision Processes, Restless Bandits	PdM
[66]	Achieving predictive and proactive maintenance for high-speed railway power equipment with LSTM-RNN	LSTM-RNN, DL, Sample Generator, Physical Degradation modeling	PdM, PsM
[132]	Real-time hierarchical neural network based fault detection and isolation for high-speed railway system under hybrid AC/DC grid	Hierarchical Neural Networks, GRU, LSTM, FPGA-based Real-Time Systems	PdM
[110]	Rail weld defect prediction and related condition-based maintenance	Extreme Learning Machine (ELM), Random Forest, Logistic Regression, PCA, SVM	PdM (CBM)
[136]	Anomaly detection of trackside equipment based on GPS and image matching	GPS Matching, Image Processing, Anomaly Detection	PdM
[111]	SCueU-Net: Efficient damage detection method for railway rail	SCueU-Net, Deep Learning, Image Segmentation	PdM
[112]	Broken rail prediction with machine learning-based approach	Extreme Gradient Boosting (XGBoost), Feature Importance Analysis, AUC Evaluation	PdM
[137]	Prediction of railway switch point failures by artificial intelligence methods	ANN, SVM	PdM
[113]	Railway turnout system RUL prediction based on feature fusion and genetic programming	Feature Fusion, AAKR, Genetic Programming	PdM
[114]	On Applying Machine Learning and Simulative Approaches to Railway Asset Management	K-means, SVM, Petri Nets, Bayesian Networks, MILP, DSS	PdM
[115]	Application of machine learning methods for predicting hazardous failures of railway track assets	Decision Trees, Random Forest, Logistic Regression, SVM	PdM
[116]	Rail breaks arrival rate prediction: A physics-informed data-driven analysis for railway tracks	Physics-Informed Machine Learning, Weibull Distribution, Bayesian Inference	PdM
[138]	Unsupervised machine learning techniques to prevent faults in railroad switch machines	K-Means, DBSCAN, PCA, Clustering Analysis	PdM
[117]	Monitoring System for Railway Infrastructure Elements Based on Thermal Imaging Analysis	Thermal Imaging, SVM, CNN, Image Processing, Expert Systems	PdM
[84]	Rail Defect Measurement System: Integrating AI and IoT for Predictive Operations	CNN, LSTM, SVM, IoT sensors, cloud computing, edge AI, ultrasonic and vibration analysis	PdM
[118]	Scheme Design of Railway Predictive Maintenance Based on IoT and AI Technology	IoT-based architecture, Priority matrix, Smart Sensors	PdM
[119]	Implementation of Artificial Intelligence for Maintenance Operation in the Rail Industry	AI, Smart Sensors	PdM
[122]	A deep reinforcement learning approach for rail renewal and maintenance planning	Deep Reinforcement Learning (DDQN), Prioritised Replay, Cox Hazard Model	PdM (CBM)
[133]	A New Approach Based on Predictive Maintenance Using Fuzzy Classifier	Fuzzy Logic, IoT, Computer Vision	PdM
[13]	Railway Digital Twins and AI: Challenges and Design Guidelines	DT, ML, Blockchain, IoT	PdM
[120]	Big-data driven assessment of railway track and maintenance efficiency using Artificial Neural Networks	ANN, ML, Big Data, Tamping Efficiency	PdM
[121]	Prediction of Railway Track Condition for Preventive Maintenance by Using a Data-Driven Approach	Logistic Regression, PCA, Data-Driven	PvM, PdM
[90]	An Architecture for Predictive Maintenance Using 3D Imaging: A Case Study on Railway Overhead Catenary	LiDAR-based 3D Point Cloud Data (PCD), DT, Microservices Architecture, DL, Distributed Computing	PdM
[123]	Leveraging AI and Deep Learning for Predictive Rail Infrastructure Maintenance	DL, ANN, LSTM, CNN	PdM
[146]	Railway infrastructure maintenance efficiency improvement using deep reinforcement learning integrated with digital twin	Deep Reinforcement Learning (A2C), Digital Twin	PdM
[129]	DAYDREAMS - Development of Prescriptive Analytics Based on Artificial Intelligence for Railways Intelligent Asset Management Systems	Artificial Intelligence, ML, Multi-Objective optimization, Prescriptive Analytics, Context-driven Human–Machine Interface (HMI), Blockchain	PsM
[147]	Predictive-Cognitive Maintenance for Advanced Railway Management	TinyML, Edge Computing, DT, MEMS	PdM
[124]	Innovative Approaches in Railway Management: Leveraging Big Data and Artificial Intelligence for Predictive Maintenance of Track Geometry	Statistical analysis (Kolmogorov–Smirnov, Welch t-tests), regression models, ANN	PdM
[92]	An AI-based Method for Predictive Maintenance of Railway Radio Communication Systems	FPCA, DTW, Supervised ML, Ensemble Classifiers	PdM
[93]	An Artificial Intelligence Approach for Automated Asset Management of Railway Systems	CNN, Feature Engineering, RUL Estimation, Diagnostic Train Data	PdM
[125]	Autonomous Maintenance in Railways using AI Techniques	ANN, DL	PdM
[128]	A GCN for optimal intelligent predictive maintenance of railway tracks	GCN, GraphSAGE, DL, Optimización (MIP)	PdM, PsM
[65]	Advancing Rail Infrastructure: Integrating Digital Twins and CPS for Predictive Maintenance	Digital Twin, CPS, IoT, AI, Edge/Cloud, RL	PdM
[126]	Design of Sensorised Rail Pads for Real-Time Monitoring and Predictive Maintenance of Railway Infrastructure	Linear Regression, ANN possible extension	PdM
[89]	Implementation of an AI-based predictive structural health monitoring strategy for bonded insulated rail joints using digital twins under varied bolt conditions	Finite Element modeling (FEM), DT, supervised ML classifiers, MATLAB Classification Learner	PdM
[127]	Machine learning. Augmented digital twin systems for predictive maintenance in high-speed rail networks	Digital Twin, RL, CNN, Autoencoders, Edge Computing, Federated Learning, Multi-Agent Systems	PdM
[148]	Real-time learning for real-time data: online machine learning for predictive maintenance of railway systems	Online ML, Concept Drift Adaptation, Streaming Pipelines, Real-time Monitoring, Anomaly Detection	PdM
[149]	Prediction of Rail Contact Fatigue on Crossings Using Image Processing and Machine Learning Methods	Magnetic Particle Inspection (MPI), High-Resolution Photo Inspection (HRPI), Image Processing, Principal Component Analysis (PCA), Polynomial Regression	PdM
[150]	A Novel Approach to Railway Track Faults Detection Using Acoustic Analysis	Acoustic Signal Analysis, MFCC Features, Logistic Regression, SVM, Random Forest, Decision Tree, MLP, CNN	PdM
[117]	Monitoring System for Railway Infrastructure Elements Based on Thermal Imaging Analysis	Thermal Imaging, Image Processing, SVM, CNN, Expert System, Conversational Interface	PdM

## Data Availability

The original contributions presented in this study are included in the article. Further inquiries can be directed to the corresponding author.

## Abbreviations

The following abbreviations are used in this manuscript:

**Abbreviation**	**Meaning**
A2C	Advantage Actor–Critic
AAKR	Auto-Associative Kernel Regression
ABA	Axle Box Accelerometer
AC	Alternating Current
ADASYN	Adaptive Synthetic Sampling
ADIF	Administrador de Infraestructuras Ferroviarias (Spanish IM)
ADP	Adaptive Dynamic Programming
AI	Artificial Intelligence
ANN	Artificial Neural Network
AR	Augmented Reality
AUC	Area Under the Curve
BERT	Bidirectional Encoder Representations from Transformers
BIM	Building Information Modeling
BSB	Bitumen-Stabilized Ballast
CAGR	Compound Annual Growth Rate
CBM	Condition-Based Maintenance
CC	Control–Command
CCIS	Control Command Information System
CCS	Control–Command and Signaling
CDE	Common Data Environment
CIRP	International Academy for Production Engineering
CNN	Convolutional Neural Network
CPS	Cyber–Physical Systems
CV	Computer Vision
CVF	Computer Vision Foundation
DAYDREAMS	EU Project: Prescriptive Analytics for Intelligent Asset Management
DBSCAN	Density-Based Spatial Clustering of Applications with Noise
DDQN	Double Deep Q-Network
DIN	Deutsches Institut für Normung
DL	Deep Learning
DRL	Deep Reinforcement Learning
DSS	Decision Support System
DT	Digital Twin
DTS	Digital Twin System
DTW	Dynamic Time Warping
EC3	Eurocode 3 (Structural Design Standard)
ELM	Extreme Learning Machine
EMR	Electromagnetic Radiation
EN	European Norm (European Standard)
ERA	European Union Agency for Railways
ERM	Empirical Risk Minimization
ERTMS	European Rail Traffic Management System
EU	European Union
EWSHM	European Workshop on Structural Health Monitoring
FEM	Finite Element Method
FFT	Fast Fourier Transform
FP	False Positive
FPCA	Functional Principal Component Analysis
FPGA	Field Programmable Gate Array
FRMCS	Future Railway Mobile Communication System
GAI	Generative Artificial Intelligence
GAN	Generative Adversarial Network
GBM	Gradient Boosting Machine
GCN	Graph Convolutional Network
GIS	Geographic Information System
GM	Grey Model
GP	Genetic Programming
GPS	Global Positioning System
GRU	Gated Recurrent Unit
GSM	Global System for Mobile Communications
GSM-R	Global System for Mobile Communications for Railway
HMI	Human–Machine Interface
HRPI	High-Resolution Photo Inspection
HSR	High-Speed Rail
ICT	Information and Communication Technology
ILP	Inductive Logic Programming
IM	Infrastructure Manager
IP	Internet Protocol
IoT	Internet of Things
ITSC	Intelligent Transportation Systems Conference
ITMC	International Transportation Management Conference
JAS	Journal of Applied Sciences
JCE	Journal of Civil Engineering
JQME	Journal of Quality Measurement and Evaluation
KDE	Kernel Density Estimation
kNN	k-Nearest Neighbours
LDA	Linear Discriminant Analysis
LIME	Local Interpretable Model-Agnostic Explanations
LLM	Large Language Model
LSTM	Long Short-Term Memory
LTE	Long-Term Evolution
MATLAB	MATLAB Technical Computing Environment
MDP	Markov Decision Process
MFCC	Mel-Frequency Cepstral Coefficients
MILP	Mixed Integer Linear Programming
MIP	Mixed-Integer Programming
MIT	Massachusetts Institute of Technology (verify context)
ML	Machine Learning
MLP	Multilayer Perceptron
MMS	Maintenance Management System
MPI	Magnetic Particle Inspection
MPLS	Multiprotocol Label Switching
MTITS	Modern Trends in Intelligent Transportation Systems (Conference)
NDT	Non-Destructive Testing
OLE	Overhead Line Equipment
PAC	Probably Approximately Correct (Learning Framework)
PCA	Principal Component Analysis
PCD	Point Cloud Data
PdM	Predictive Maintenance
PHM	Prognostics and Health Management
PRISMA	Preferred Reporting Items for Systematic Reviews and Meta-Analyses
PsM	Prescriptive Maintenance
PvM	Preventive Maintenance
R2F	Run-to-Failure
RCF	Rolling Contact Fatigue
RDMS	Rail Defect Measurement System
REINFORCE	Monte-Carlo Policy Gradient Method
RFIG	Red Ferroviaria de Interés General
RIS	Reconfigurable Intelligent Surfaces
RL	Reinforcement Learning
RNN	Recurrent Neural Network
RNNLSTM	Recurrent Neural Network with LSTM units
RMSE	Root Mean Square Error
RUL	Remaining Useful Life
SCueU-Net	Saliency Cue U-Net (Segmentation Model)
SDN	Software Defined Networking
SGS	Safety Management System
SHAP	SHapley Additive exPlanations
SNE	Stochastic Neighbor Embedding
SOM	Self-Organizing Map
SVM	Support Vector Machine
SVR	Support Vector Regression
TCN	Temporal Convolutional Network
THI	Track Health Index
TQI	Track Quality Index
TSI	Technical Specifications for Interoperability
TSM	Technical Safety Management
UIC	International Union of Railways
VC	Vapnik–Chervonenkis (Dimension)
VNS	Variable Neighborhood Search
VR	Virtual Reality
XAI	Explainable Artificial Intelligence
